# An updated national survey of triage and triage related work in Sweden: a cross-sectional descriptive and comparative study

**DOI:** 10.1186/s13049-021-00905-2

**Published:** 2021-07-03

**Authors:** Sara C. Wireklint, Carina Elmqvist, Katarina E. Göransson

**Affiliations:** 1 Emergency Department and Department of Research and Development, Region Kronoberg, Department of Health and Caring Sciences and Centre of Interprofessional Collaboration within Emergency Care (CICE), Linnaeus University, FoU Kronoberg, Sigfridsvägen 5, S-352 57 Växjö, Sweden; 2 Department of Research and Development, Region Kronoberg and Centre of Interprofessional Collaboration within Emergency Care (CICE) at the Department of Health and Caring Sciences, Linnaeus University, Växjö, Sweden; 3 Department of Medicine Solna, Karolinska Institutet and Emergency and Reparative Medicine Theme, Karolinska University Hospital, Stockholm, Sweden

**Keywords:** Emergency service, hospital – emergency department, Education – competency-based education, Health care quality, access, and evaluation – cross-sectional study, Rapid emergency triage and treatment system, Sweden, Triage – emergency medical service

## Abstract

**Background:**

Triage and triage related work has been performed in Swedish Emergency Departments (EDs) since the mid-1990s. According to two national surveys from 2005 to 2011, triage was carried out with different triage scales and without guidelines or formal education. Furthermore, a review from 2010 questioned the scientific evidence for both triage as a method as well as the Swedish five level triage scale Medical Emergency Triage and Treatment System (METTS); nevertheless, METTS was applied in 65% of the EDs in 2011. Subsequently, METTS was renamed to Rapid Emergency Triage and Treatment System (RETTS©). The hypothesis for this study is that the method of triage is still applied nationally and that the use of METTS/RETTS© has increased. Hence, the aim is to describe the occurrence and application of triage and triage related work at Swedish Emergency Departments, in comparison with previous national surveys.

**Methods:**

In this cross-sectional study with a descriptive and comparative design, an electronic questionnaire was developed, based on questionnaire from previous studies. The survey was distributed to all hospital affiliated EDs from late March to the middle of July in 2019. The data was analysed with descriptive statistics, by IBM SPSS Statistics, version 26.

**Results:**

Of the 51 (75%) EDs partaking in the study, all (100%) applied triage, and 92% used the Swedish triage scale RETTS©. Even so, there was low concordance in how RETTS© was applied regarding time frames i.e., how long a patient in respective triage level could wait for assessment by a physician. Additionally, the results show a major diversion in how the EDs performed education in triage.

**Conclusion:**

This study confirms that triage method is nationally implemented across Swedish EDs. RETTS© is the dominating triage scale but cannot be considered as one triage scale due to the variation with regard to time frames per triage level. Further, a diversion in introduction and education in the pivotal role of triage has been shown. This can be counteracted by national guidelines in what triage scale to use and how to perform triage education.

**Supplementary Information:**

The online version contains supplementary material available at 10.1186/s13049-021-00905-2.

## Background

In recent decades, problems with overcrowded Emergency Departments (EDs) have become a common and well-known issue all over the world [1], where Sweden is no exception. In order to handle the problem, the method of triage has been implemented from the military [2, 3] and several triage scales have been developed since the 1960s. A triage scale is used when performing triage, usually one with five levels. The most commonly known are the Australasian Triage Scale (ATS) [4], the Canadian Triage and Acuity Scale (CTAS) [5], the Manchester Triage System (MTS) in the UK [6], the Emergency Severity Index (ESI) [7] and the South African Triage Scale (SATS) [8, 9].

Publications around ED triage in the Scandinavian countries are found from early 2000. The first study we have been able to find is based on data from Swedish EDs in the mid-1990s [10]. According to this study, based on 70/81 (86%) of the Swedish EDs, some kind of triage or triage related work was performed in the mid-1990s. Further, triage was performed by registered nurses (RNs) in 38 (54%) of the EDs, but, with deficient support such as formal education or written guidelines in 21 of these 38 EDs [10]. A follow-up survey was performed in the mid-2000s, with a response rate of 69/79 (87%). This study showed that 78% of the EDs were familiar with the concept of triage/priority, and that one-third (24/69) had a designated RN in triage. In 37 EDs some kind of triage scale was applied but there was no consistency regarding which triage scale to use. Instead several different triage scales were employed, with triage levels ranging from three to five levels, and with diverging time frames for all levels except the most acute level. There were also various methods to express acuity; numeric rating, however, was the most common practice [11]. The most recent Swedish study (response rate 100%) showed that 72 EDs (97%) of all EDs applied triage. Almost 88% used a five-level triage scale. Most common was the Swedish Medical Emergency Triage and Treatment System (METTS) (65%), followed by another Swedish triage scale, Adaptive Process Triage (ADAPT) (19%) and MTS (4%). Locally developed scales were applied in 9% of the EDs [12].

A similar progression of triage implementation has been shown in the other Scandinavian countries, where it has been studied. A Danish study, based on 95% of the EDs and published 2011, showed that the method of triage was used at 75% of the EDs, and in 73% triage was performed by a RN. ADAPT was the most commonly used (25%) validated triage scale, but MTS and ESI was also applied (10% each). However, the majority (40%) of the EDs used non-validated systems [13]. In Norway, a study published 2013 and based on 80% of all EDs, showed that 100% applied triage. Of these, 76% used a triage scale, and 50% of these used an established five-level triage scale. MTS was the most common, but also METTS and CTAS was applied. In 25 of 45 EDs the triage was performed by a RN [14]. According to these two studies, triage has been applied since 2004 in Norway and 2009 in Denmark [13, 14].

In 2010 The Swedish Council on Health Technology Assessment published a systematic review regarding triage and patient flow processes. The review concluded that there was a low scientific foundation for triage as a method, and deficient scientific foundation for the Swedish triage scale METTS [15], which subsequently became Rapid Emergency Triage and Treatment System (RETTS©) and copyrighted in 2011 [16]. In summary; considering the history of triage development described in Scandinavia in general and Sweden in particular, it can be assumed that the application of triage and triage scales has continued, despite the low scientific foundation [15]. The hypothesis of present study is therefore that the method of triage is still applied nationally and that the use of METTS/RETTS© has increased.

## Methods

### Aim

To describe the occurrence and application of triage and triage related work at Swedish Emergency Departments, in comparison with previous national surveys.

### Design

The study has a cross-sectional descriptive and comparative design.

### Setting and materials

Inclusion criteria for the study was hospital affiliated ED in Sweden (*N* = 68) [17]. Exclusion criteria was EDs with less than two co-located somatic specialties. Furthermore, the hospitals are classified into three categories regarding competencies; county, regional and university hospital. The county hospital has on average 12 to 13 medical areas of activity, the regional 23 and the university hospital 40. The university hospitals performs highly specialized medical care with a national intake [18]. In Sweden, the majority of the RNs hold a bachelors’ degree in nursing, and a specialist RN often hold a one-year master degree. Specific formal education in ED triage, is limited to those undergoing the Emergency Nursing Specialist Program.

A questionnaire (Additional file 1) was produced for the survey. Since the questionnaires from the two previous Swedish surveys [11, 13] were found insufficient related to the number of questions [13] and outdated formulations [11], a new questionnaire was produced. However, the new questionnaire originates from previous questionnaires as well as the results from those studies [11, 13]. The questionnaire was pilot tested twice for face validity by a total of five persons, four head of departments and one party responsible for triage at that particular ED. These respondents answered the survey’s 30 items, and 12 questions about the construction of the survey. The first pilot test performed by two respondents yielded some corrections. The second pilot, performed by the remaining three respondents, did not result in any further changes. However, the idea of making the survey electronic was suggested by one of these respondents. The electronic survey instrument esMaker was therefore applied to the survey. The 30 items on the final survey contained mostly close-ended questions in combination with the possibility to add information.

### Data collection/process

All operational managers or head of the department for the EDs were contacted by phone by the first author. Information about the study was given together with an invitation to participate; all approved the study. One or 2 days after the phone-call, the survey was distributed by e-mail. The survey was mainly answered by persons in the managerial position (59%), and thereafter by RNs (37%), often with education or RETTS/triage responsibility. One survey (2%) was answered by a physician. Three reminders were sent with a 10-day interval, and 10 days after the third reminder the survey was closed. The data collection was performed over less than 4 months, from March 27th, to July 13th 2019. A completed survey was considered as a written consent. All data were collected by the first author.

### Analysis

Descriptive statistics were carried out using IBM SPSS Statistics, version 26.

## Results

All 68 EDs in Sweden accepted to participate and 51 (75%) completed the survey. All of the responding EDs (100%) applied the triage method, and the Swedish triage scale RETTS© was the most commonly used triage scale (92%) (Table 1).

**Table 1:** Participating EDs and triage scales in use in Swedish EDs 51/68 (75%)

**The type of hospital, n (%)**	
*County* ^*A*^	28 (55)
*Regional* ^*B*^	16 (31)
*University* ^*C*^	7 (14)
TOTAL	51 (100)
**Triage scales, n (%)**	
*RETTS©*^*a*^	47 (92)
*SATS*^*a*^	2 (4)
*Locally developed*^*a*^	1 (2)
*No name*^*b*^	1 (2)
TOTAL	51 (100)

^A^ *N* = 35; ^B^ *N* = 22; ^C^ *N* = 11

^a^ Five-level triage scale ^b^ Six-level triage scale

The participating hospitals were representative of the Swedish context, and in 11 of 22 regions (50%), there was 100% participation of the EDs. The attrition rate was 15% (9/61), and there was a total of 31 answers missing, from 17 different EDs.

### Occurrence of triage

The majority of the EDs (63%) declared that the main purpose for triage was to establish order of clinical urgency. Walk-in patients were triaged in all EDs while patients arriving by ambulances were triaged in 37 (72%) EDs. In 49 (96%) EDs, the same triage scale was also applied in the pre-hospital setting. Triage was applied 24 h a day, 7 days a week in 46 (90%) of the ED. Furthermore, in 50 EDs (98%) triage was performed by a RN, with or without a specialist degree. In the majority, (82%) the RN worked with assistant nurses (ANs) or some other personal category forming a triage team. In three EDs (6%) physicians were involved in performing the triage at some time during the day, together with a RN and an AN. One ED reported that other personnel categories in the triage. The described staffing was the same 24 h a day in 39 (76%) EDs.

### Triage application

**Fig. 1:**
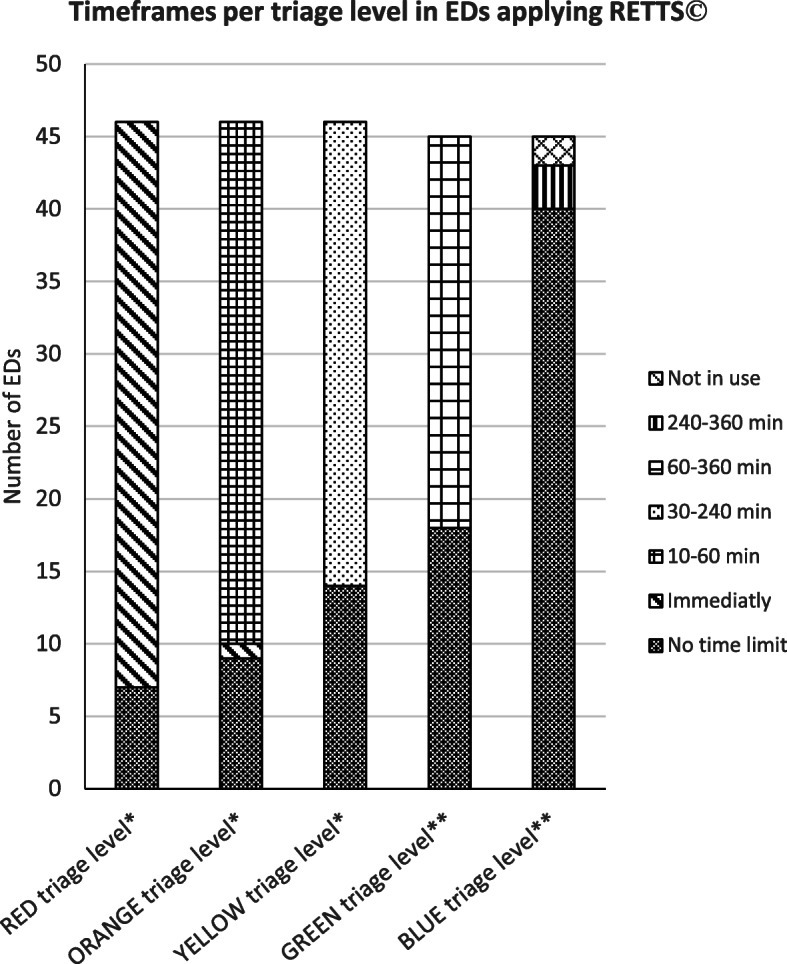
time frames per triage level applied by the 47 EDs using RETTS©. * One missing. ** Two missing

In order to perform triage, all 51 (100%) EDs reported that a triage scale of any kind was applied (Table 1). Time frames, i.e. the time a patient is assessed to be able to wait at respective triage level, for assessment and treatment by a physician, without risk for medical deterioration, was applied in 44 (86%) of the EDs. The EDs that applied RETTS© reported varying time frames in all triage levels (Fig. 1).

In the red triage level, a two-fold diversion was found. Seven different time frames were observed within the orange triage level, in the figure merged into three groups. The most common was 15 min (28%). In both yellow and green triage levels, ten different time frames were reported per level, which, in the figure, are presented merged into two major groups per triage level. The most common time frame in the yellow triage level was 120 min (41%), and 240 min (42%) in the green one. The blue triage level was attached to 12 different time frames, merged into three groups in the figure. The two hospitals that applied SATS had no in-between difference regarding time frames per triage level; they ranged from immediately (red) to no time limit (blue). The locally developed triage scale applied time frames that ranged from immediately to 240 min. Twelve (23%) of the EDs reported that they excluded the triage level with the lowest acuity rank, i.e. in practice they applied triage with a four-level triage scale. In 50 (98%) of the EDs, colours were used to mark triage levels, while one ED used colour in combination with numbers.

### Triage; process and interventions

**Fig. 2:**
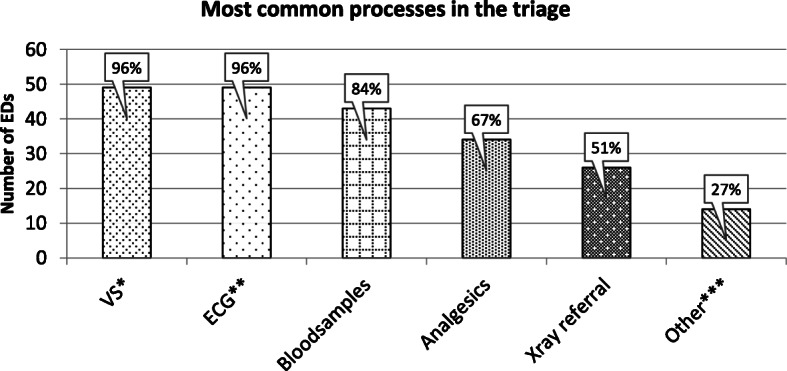
Processes performed in the triage. * VS = vital signs. ** ECG = electrocardiogram. *** Other = for example peripheral intravenous treatment, urine sample, bladder scan, oxygen administration, wound dressing, counselling, pain assessment, treatment of allergic reaction etc.

All the EDs declared that they performed processes of some kind during triage (Fig. 2).

The majority (96%) performed one or more processes in combinations. Most common was the combination of five processes; blood sample, ECG, X-ray referral, analgesics and VS (27%). Two EDs declared that they did not have a specific triage team, therefore they just answered other processes without specifying what. Seven (14%) of the EDs declared that they did all five specified interventions as well as the non-specified, i.e. other intervention.

**Fig. 3:**
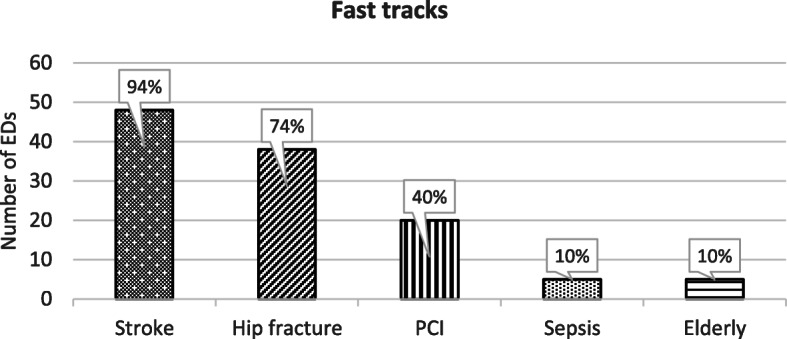
Fast tracks applied as reported by 50 EDs

The intervention fast track, i.e. a special, coherent process for a specific patient/diagnosis, of some kind was performed in 50 (98%) of the EDs (Fig. 3).

The majority (88%) of the EDs applied more than one fast track, most commonly two fast tracks in different combinations (48%). The most common combination was the stroke and the hip fracture, which was performed in 16 (31%) of the EDs, followed by the combination of three fast tracks; the stroke, the hip fracture, and the PCI, reported by eight (16%) EDs.

### Triage education

In 44 EDs (86%) triage education was performed mainly as basic education (84%) and mostly during the introduction of new employees (39%). Refreshment courses were applied in 27 (53%) of the EDs. The education was usually executed by the persons with triage responsibility on the ED (57%). In one third of the EDs the education was theoretical, and in seven EDs the education was founded on a combination of theory and practice. The time spent on education varied with a continuum ranging from 15 min to 2 weeks; the most common practice was between 30 min to 2 h (36%). One ED applied three-day training alongside a colleague.

## Discussion

The result of the study is based on 51 (75%) Swedish EDs, and confirms the hypothesis that triage in general, and the RETTS© triage scale in particular, has become firmly implemented. Furthermore, the result shows the progression in the use of processes and interventions when triage is carried out, together with introduction and education in triage.

The present study shows an increase in the application of RETTS© compared to the findings of Farrokhnia et al. [12]. The result also reveals that ADAPT and MTS that was in use in 2011 seems to have been replaced by SATS. A similar pattern can be seen in Norway, where RETTS© and a version of SATS, called SATS Norway, have become more commonly used, even if MTS is still applied in some extent [19]. However, ADAPT have been triage standard in a modified version called Danish Emergency Process Triage (DEPT) in Denmark since 2011 [20]. A version of RETTS©, called Rapid Emergency Triage and Treatment System – Hospital Unit West (RETTS-HEV) has also been applied and studied [21, 22]. Furthermore, a new, simplified triage algorithm has been developed in Denmark called the Copenhagen Triage Algorithm (CTA). CTA is quite similar to ADAPT/DEPT but is based on clinical assessment and cut-off point for vital signs [23], which are calculated on results from the TRIAGE database [24]. In a cluster-randomized study, CTA has been found to be non-inferior to ADAPT [25]. Both Göransson et al. [11] and Farrokhnia et al. [12] reported the use of locally developed triage scales; this approach still exists, but to a lesser extent, as in Norway [19]. Internationally the use of locally developed scales is mostly reported in comparison to more established triage scales like ESI and MTS [26].

An alteration regarding triage according to the arriving mode was found; previous study reported triaging 100% of the patients arriving by an ambulance [11], in contrast to 72% in present study. This might be explained by the fact that the same triage scale was used both in the pre-and intra-hospital setting in 96% of the EDs. However, according to Domagala and Vets (2015) all patients should be triaged and treated corresponding to their medical condition, regardless of their mode of arrival [27]. Additionally, the level of medical competence in the triage situation has increased; in 98% of the EDs triage is carried out by a RN, with or without a specialist degree, in contrast to about 50% reported previous [10, 11], as well as in Norway [19]. Interestingly, a study from Denmark discuss that clinical assessment can be applied through an eye-ball triage, which can be performed by hospital staff without formal training in patient evaluation or experience of formalised triage [20].

Farrokhnia et al. [12], as well as Stadheim Halvorsen et al. [19], discussed the benefit of having a mutual triage scale which could enable a common language, thereby facilitating understanding for the patient’s acuity in and pre- and intra hospital context. However, such potential effects require a common, valid and reliable triage scale. Even though RETTS© has been widely implemented across Sweden, this study questions whether it is a single, unified triage scale. Furthermore, there is, to the best of our knowledge, a lack of studies on RETTS© in Swedish context. The latest validity study was published in 2011 [28] based on data from 2006, and the most recent reliability study demonstrated just a moderate inter-reliability [29]. Nevertheless, studies in Denmark have found RETTS-HEV to have high predictive validity [22] and good overall inter-rater agreement [21], and RETTS-paediatric version has been found to have high inter- and intra-reliability [30] and high validity [31] in Norway. However, the demonstrated diversion regarding time frames can result in further confusion rather than better understanding. The diversion can be explained by the fact that RETTS© does not stipulate any time frames [16], yet a previous study has showed that it can be difficult to establish concordance in the triage assessments even with the same time frames and education [29]. Further, the present study reveals conformity regarding the presentation of triage levels; all EDs use the same colour code; red for the most acute, followed by orange, yellow, green and blue; one ED combined these colours with numbers. This is positive progress compared with the five different ways to communicate acuity indicated by Göransson et al. [11].

The results of this study show that several processes and interventions discussed in earlier studies are applied. Göransson et al. [11] observed that the examinations during triage varied from merely taking the patients’ chief complaint to planning the blood samples and X-rays, which is what Palmqvist and Lindell had already discussed 20 years ago [10]. Farrokhnia et al. [12] showed that a number of interventions were implemented or planned to be implemented such as a nurse required X-ray (59% of the EDs), fast track (47%) and team triage (43%). However, the result of this study shows that ECG, taking blood sampled and giving analgesics are more commonly performed than referral to an X-ray. Nevertheless, on the other hand, the fast track, already mentioned by Palmqvist & Lindell [10], is now implemented in 98% of the EDs. Regarding team triage, Farrokhnia et al. [12] didn’t present any definition of the concept, but Oredsson et al. [32] defines it as a team in the triage that includes a physician. Two recent studies [33, 34] indicate a significant quality improvement in time to physician with physician-led team triage compared with RN-led triage. Nevertheless, one of these authors concludes in her thesis that the positive outcomes are not sustained over time [35]. However, if the combination of a RN with an AN is considered to be a team, it can be concluded that 82% of the EDs has implemented this intervention, which is more than expected when compared with Farrokhnia et al. [12].

Unfortunately, the lack of written guidelines described by Palmqvist & Lindell [10] and Göransson et al. [11] is still present, especially regarding triage education. This may imply varying levels of competence of the RN during triage and contradicts the demonstrated fact that it is vital to have high competence and skills when performing triage [36]. Since there is no evidence that experienced RNs perform better, in the sense of inter-rater reliability during triage [37–39], education is crucial [40]. The Emergency Nurses Association therefore declares that evidence based, regular education is required to maintain quality and safety [41]. In summary, the results regarding education presented in this study describes a complex image of how education in triage is applied in Sweden today, and it appears that a mutual platform on how to perform triage education is lacking.

### Strengths and limitations

The construction of the current study is based on the study conducted by Göransson et al. [11]. The facial certification of this new structure has been tested twice in pilot tests. Both tests yield good results, both for the survey and questionnaire. Furthermore, the main strength of the study, is the high response rate. However, the main limitation is that almost 30% of the missing surveys were distributed to EDs with high inflow, i.e. regional/university hospitals, which might affect generalizability.

## Conclusions

ED triage can be viewed as nationally implemented in Sweden but there is a lack of a nationally uniform triage scale. The RETTS© scale is the dominating triage scale but, due to the lack of standardized time frames per triage level, cannot be considered one ED triage scale. Further, a diversion regarding how RNs are introduced and educated in the pivotal role of triage has been shown. In order to counteract this, national guidelines should be introduced regarding what triage scale to use, how to use it and how to perform triage education, as a conceivable approach to handling the problem.

## Supplementary Information

**Additional file 1.**

## Data Availability

The dataset used and analysed during the present study are available from the corresponding author on reasonable request.

## Abbreviations

ED: Emergency department
RN: Registered nurse
AN: Assistant nurse
ECG: Electrocardiogram
VS: Vital signs
