# Supplementary material for: An updated national survey of triage and triage related work in Sweden: a cross-sectional descriptive and comparative study
Source: Scand J Trauma Resusc Emerg Med. 2021 Jul 3;29:89. doi: 10.1186/s13049-021-00905-2 (PMC8254961; doi:10.1186/s13049-021-00905-2)
Supplement: Supplementary file 1 — Additional file 1. [file 13049_2021_905_MOESM1_ESM.docx]

|  | 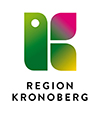 |
| --- | --- |

Description of activity

**1. Your position/function/area of responsibility is**

| 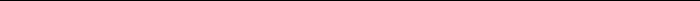 |
| --- |

**2. Please indicate the number of patients visit your Emergency Department had in 2018 excluding those who were referred**

| 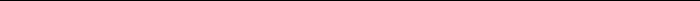 |
| --- |

**3. Which patient groups are being cared for in your emergency department?  Multiple choices are possible**

| 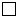 | Children |  |
| --- | --- | --- |
| 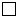 | Adult |  |
|  | Specify any limitations, e.g. if not all children are taken care of, what is the limit, clinic, age, other? |  |
|  | 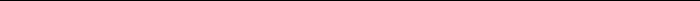 | |
|  |  | |

**4. What medical specialties do you have access to in your emergency department? Multiple choices are possible**

| 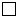 | Emergency medicine/emergency physician |  |
| --- | --- | --- |
| 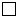 | Children |  |
| 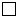 | Gynaecology |  |
| 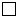 | Infection |  |
| 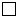 | Surgery |  |
| 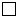 | Medicine |  |
| 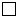 | Orthopaedics |  |
| 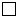 | Eyes |  |
| 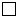 | Ear Nose Throat |  |
| 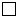 | Other |  |
|  | 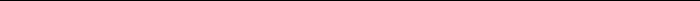 | |

**5. Are there limitations in the above? For example, some specialties only available as an on-call function**

| 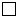 | YES, please indicate which ones and describe how |  |
| --- | --- | --- |
|  | 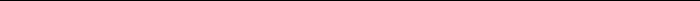 | |
| 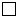 | NO |  |

Organisational description

**6. For what purpose is triage used in your emergency department? Select the overall purpose**

| 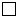 | Identifying patients suitable for referral to other levels of care |  |
| --- | --- | --- |
| 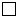 | Creating an order of priority to physician assessment |  |
| 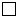 | Other, specify what |  |
|  | 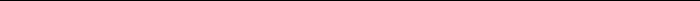 | |

**7. Which patients are triaged? Select which ones, multiple choices are possible**

| 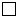 | Arrivals by ambulance |  |
| --- | --- | --- |
| 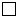 | Walk-ins Those who “walk” in |  |
| 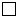 | Arriving with referral from another health care facility |  |
|  | If any of the above patient groups are excluded from undergoing triage, describe how you determine the order of priority of these patients |  |
|  | 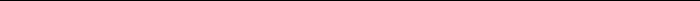 | |

**8. Is ED triage carried out 24 hours a day, 7 days a week?**

| 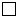 | YES |  |
| --- | --- | --- |
| 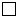 | NO, describe how your institution does this |  |
|  | 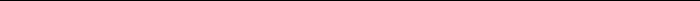 | |
|  | 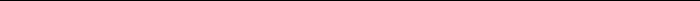 | |

**9. Which health care professionals staff the triage area? Multiple choices are possible**

| 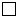 | Physicians |  |
| --- | --- | --- |
| 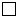 | Registered Nurses |  |
| 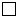 | Registered Nurses with a specialist degree |  |
| 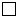 | Assistant nurse |  |
| 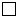 | Other |  |
|  | 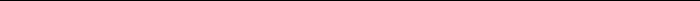 | |

**10. Does the above staffing apply around the clock?**

| 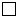 | YES |  |
| --- | --- | --- |
| 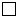 | NO, please describe how and at what time staffing differs from the above |  |
|  | 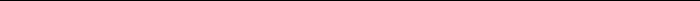 | |
|  | 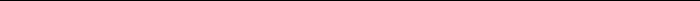 | |

Triage-specific questions

**11. Do you use a triage scale when triaging?**

| 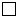 | YES, indicate the name of your triage scale and your justification for choosing this |  |
| --- | --- | --- |
|  | 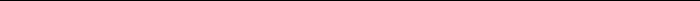 | |
| 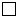 | NO |  |

**12. How many triage levels does the scale have?**

| 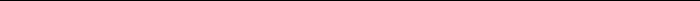 |
| --- |

**13. How are the triage levels marked?**

| 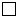 | With letters |  |
| --- | --- | --- |
| 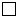 | With colours |  |
| 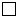 | With numbers |  |
| 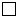 | Other means, indicate with what/how |  |
|  | 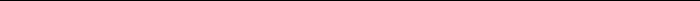 | |

**14. Describe how triage levels are ranked from most to least acute patient, e.g. red /prio 1/ XX is most acute and then in descending order**

| 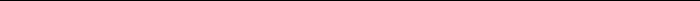 |
| --- |
| 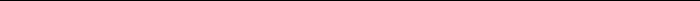 |

**15. Do you use all the scale’s triage levels? That is, all five in a five-graded, all four in a four-graded, all three in a three-graded triage scale**

| 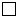 | YES |  |
| --- | --- | --- |
| 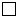 | NO |  |
|  | If NO, please indicate which level(s) is/are not used and the justification for this |  |
|  | 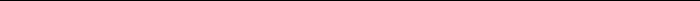 | |

**16. Is any time frame associated with the triage levels?**

| 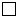 | YES |  |
| --- | --- | --- |
| 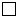 | NO |  |

**17. If YES, which ones? Enter the number of minutes per triage level, e.g. red/prio 1 - immediate, orange/prio 2 - within xx minutes, etc. Tick the box in front of each line to activate free text**

| 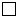 | Red/Prio 1/XX/ |  |
| --- | --- | --- |
|  | 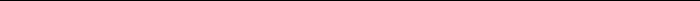 | |
| 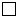 | Orange/Prio 2/XX/ |  |
|  | 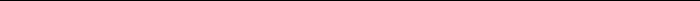 | |
| 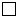 | Yellow/Prio 3/XX/ |  |
|  | 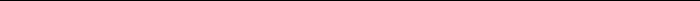 | |
| 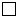 | Green/Prio 4/XX/ |  |
|  | 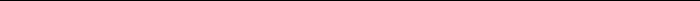 | |
| 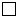 | Blue/Prio 5/XX/ |  |
|  | 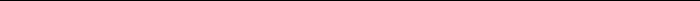 | |

**18. If you are using RETTS, in what order of priority are patients with triage level yellow, green and blue taken?**

| 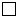 | According to triage level, e.g. yellow before green, green before blue |  |
| --- | --- | --- |
| 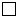 | According to arrival time, i.e. "first come first served" |  |
| 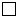 | Other order of priority |  |
| 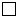 | Not using RETTS |  |
|  | If OTHER, please describe how |  |
|  | 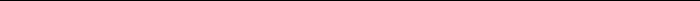 | |

**19. Are you carrying out any intervention’s actions/measures in triage? Select which ones, multiple choices are possible**

| 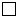 | Blood specimen collection |  |
| --- | --- | --- |
| 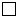 | ECG |  |
| 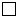 | X-ray referral |  |
| 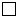 | Pain relief |  |
| 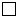 | Vital signs |  |
| 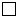 | Other |  |
|  | If OTHER, what? |  |
|  | 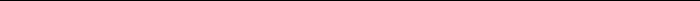 | |

**20. Do you re-assess patients who have not had time to be medically assessed within the time specified for the triage level? That is, a systematic re-evaluation**

| 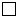 | YES |  |
| --- | --- | --- |
| 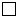 | NO |  |
|  | If YES, estimate how often; 100%? 50%? More or less? |  |
|  | 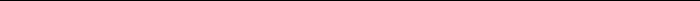 | |

**21. Is fast-track used in your emergency department? For example, fast-track cardiac, fast-track hip, fast-track stroke**

| 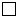 | YES, indicate which |  |
| --- | --- | --- |
|  | 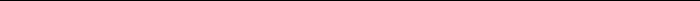 | |
| 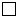 | NO |  |

**22. Is the same triage scale used in the pre-hospital setting?**

| 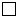 | YES |  |
| --- | --- | --- |
| 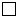 | NO |  |
| 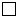 | Other triage scale, specify which |  |
|  | 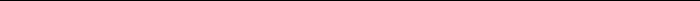 | |
| 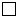 | No triage scale is used |  |

**23. Do you have any guideline regarding maximum waiting time from arrival at the emergency department up to triaging?**

| 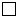 | YES |  |
| --- | --- | --- |
| 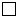 | NO |  |

**24. If YES specify Tick the box to activate free text**

| 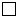 | What are the limits? |  |
| --- | --- | --- |
|  | 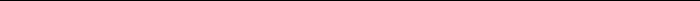 | |
|  | 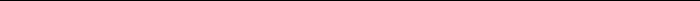 | |
| 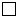 | When the limit is reached, please describe what actions you take |  |
|  | 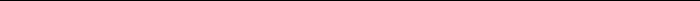 | |
|  | 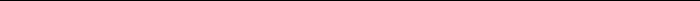 | |

Education and evaluation

**25. Is triage education provided at your emergency department? (if NO you will be transferred to question no 28)**

| 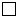 | YES |  |
| --- | --- | --- |
|  | NO |  |

**26. Select the level at which it is conducted Multiple choices are possible**

|  | Basic/beginners’ course |  |
| --- | --- | --- |
|  | Refresher training |  |

**27. Describe the education available Tick the box to activate free text**

|  | Who provides the education? |  |
| --- | --- | --- |
|  |  | |
|  | When is education provided? |  |
|  |  | |
|  | What does the educational content look like? |  |
|  |  | |
|  | How long a period is used/set aside? |  |
|  |  | |
|  | How often is training education carried out? |  |
|  |  | |

**28. Is there any evaluation of triage at your emergency department? (if NO you will be transferred to question no 30)**

|  | YES |  |
| --- | --- | --- |
|  | NO |  |

**29. Describe the evaluation Tick the box to activate free text**

|  | Who is responsible for the evaluation? Head of Triage Department, Department Head, Care Developer, Third Party? |  |
| --- | --- | --- |
|  |  | |
|  | Describe your set-up: is it according to a regular schedule, is it as needed or other arrangement? |  |
|  |  | |
|  | What are you evaluating? waiting time, accuracy, other? |  |
|  |  | |
|  | How is the evaluation carried out? |  |
|  |  | |
|  | How are the results used? for educational purposes, a discussion on training days, other? |  |
|  |  | |

Summary

**30. We will gratefully accept any other thoughts you may have regarding triage that the above questions have not covered.**

|  |
| --- |
|  |
|  |

Thank you for your participation!
